# Retinoic Acids Potentiate BMP9-Induced Osteogenic Differentiation of Mesenchymal Progenitor Cells

**DOI:** 10.1371/journal.pone.0011917

**Published:** 2010-07-30

**Authors:** Wenli Zhang, Zhong-Liang Deng, Liang Chen, Guo-Wei Zuo, Qing Luo, Qiong Shi, Bing-Qiang Zhang, Eric R. Wagner, Farbod Rastegar, Stephanie H. Kim, Wei Jiang, Jikun Shen, Enyi Huang, Yanhong Gao, Jian-Li Gao, Jian-Zhong Zhou, Jinyong Luo, Jiayi Huang, Xiaoji Luo, Yang Bi, Yuxi Su, Ke Yang, Hao Liu, Hue H. Luu, Rex C. Haydon, Tong-Chuan He, Bai-Cheng He

**Affiliations:** 1 Department of Orthopaedic Surgery, West China Hospital, Sichuan University, Chengdu, Sichuan, China; 2 Molecular Oncology Laboratory, Department of Surgery, The University of Chicago Medical Center, Chicago, Illinois, United States of America; 3 Key Laboratory of Diagnostic Medicine designated by the Chinese Ministry of Education and the Affiliated Hospitals, Chongqing Medical University, Chongqing, China; 4 Stem Cell Biology and Therapy Laboratory, The Pediatric Research Institute, the Children's Hospital of Chongqing Medical University, Chongqing, China; 5 School of Bioengineering, Chongqing University, Chongqing, China; 6 Department of Geriatrics, Xinhua Hospital of Shanghai Jiatong University, Shanghai, China; 7 Department of Cell Biology, The Third Military Medical University, Chongqing, China; 8 Department of Pharmacology, Chongqing Medical University, Chongqing, China; Universidade Federal do Rio de Janeiro (UFRJ), Brazil

## Abstract

**Background:**

As one of the least studied bone morphogenetic proteins (BMPs), BMP9 is one of the most osteogenic BMPs. Retinoic acid (RA) signaling is known to play an important role in development, differentiation and bone metabolism. In this study, we investigate the effect of RA signaling on BMP9-induced osteogenic differentiation of mesenchymal progenitor cells (MPCs).

**Methodology/Principal Findings:**

Both primary MPCs and MPC line are used for BMP9 and RA stimulation. Recombinant adenoviruses are used to deliver BMP9, RARα and RXRα into MPCs. The *in vitro* osteogenic differentiation is monitored by determining the early and late osteogenic markers and matrix mineralization. Mouse perinatal limb explants and *in vivo* MPC implantation experiments are carried out to assess bone formation. We find that both 9CRA and ATRA effectively induce early osteogenic marker, such as alkaline phosphatase (ALP), and late osteogenic markers, such as osteopontin (OPN) and osteocalcin (OC). BMP9-induced osteogenic differentiation and mineralization is synergistically enhanced by 9CRA and ATRA *in vitro*. 9CRA and ATRA are shown to induce BMP9 expression and activate BMPR Smad-mediated transcription activity. Using mouse perinatal limb explants, we find that BMP9 and RAs act together to promote the expansion of hypertrophic chondrocyte zone at growth plate. Progenitor cell implantation studies reveal that co-expression of BMP9 and RXRα or RARα significantly increases trabecular bone and osteoid matrix formation.

**Conclusion/Significance:**

Our results strongly suggest that retinoid signaling may synergize with BMP9 activity in promoting osteogenic differentiation of MPCs. This knowledge should expand our understanding about how BMP9 cross-talks with other signaling pathways. Furthermore, a combination of BMP9 and retinoic acid (or its agonists) may be explored as effective bone regeneration therapeutics to treat large segmental bony defects, non-union fracture, and/or osteoporotic fracture.

## Introduction

Mesenchymal progenitor cells (MPCs) are adherent marrow stromal cells that can self-renew [1] and differentiate into osteogenic, chondrogenic, and adipogenic lineages [2], [3], [4], [5], although MPCs have also been isolated from many tissues, such as adipose, skeletal muscle, periosteum, brain, liver, bone marrow, amniotic fluid and hair follicle [6], [7], [8], [9], [10], [11], [12], [13], [14], [15]. Osteogenesis is a sequential cascade that recapitulates most, if not all, of the cellular events occurring during embryonic skeletal development [16]. During skeletogenesis, bone formation occurs through two different pathways, intramembranous ossification or endochondral ossification [16]. Bone regeneration following a fracture progresses through sequential phases similar to endochondral ossification, starting with chemotaxis and proliferation of MPCs [2], [3], [4], [5].

Bone morphogenetic proteins (BMPs) play an important role in regulating cell proliferation and differentiation during development [5], [17], [18], [19] and have been shown to play an important role in stem cell biology [20], [21]. BMPs belong to the TGFβ superfamily and consist of at least 14 members in humans [5], [17], [18], [19], [22]. Genetic disruptions of BMPs have resulted in various skeletal and extraskeletal abnormalities during development [5], [22], [23]. BMPs fulfill their signaling activity by interacting with the heterodimeric complex of two transmembrane serine/threonine kinase receptors, BMPR type I and BMPR type II [17], [19]. The activated receptor kinases phosphorylate the transcription factors Smads 1, 5, or 8, which in turn form a heterodimeric complex with Smad4 in the nucleus and regulate the expression of target genes in concert with other co-activators [5], [17]. Upon analyzing the 14 types of BMPs, we found that BMP9 is one of the most potent BMPs in inducing osteogenic differentiation of MPCs [19], [24], [25], [26], [27], [28], [29], [30], [31]. We further demonstrated that BMP9 regulates a distinct set of downstream targets that may play a role in regulating BMP-induced osteoblast differentiation of MPCs [19], [26], [27], [28], [29], [31]. Although the functional role of BMP9 in skeletal system remains to be fully understood, its potent osteogenic activity suggests that it may be used as an efficacious bone regeneration agent. It is conceivable that other signaling molecules may act synergistically to enhance BMP9-induced bone formation.

Retinoic acids (RAs) play an important role in embryonic development and function maintenance of vital organs in adult [32], [33]. RAs regulate differentiation and metabolism by serving as ligands for two families of nuclear receptors, the RA receptors (RARα, RARα, and RARγ) that bind the abundant form of RA known as all-trans-RA (ATRA) and the retinoid X receptors (RXRα, RXRβ, and RXRγ) that bind an isomer known as 9-cis-RA (9CRA) [34], [35], normally undetectable except when vitamin A is present in excess [36]. RA binding to RAR/RXR heterodimers bound to a regulatory DNA element leads to a cascade of events resulting in recruitment of transcriptional co-activators and initiation of transcription [35], [37]. Genetic manipulations in animals have revealed that RA signaling is important for the development of the forebrain and the segmented hindbrain, and for the elongation of the body axis [32], [33], [35]. Agonists of RAR, and RXR have been shown to promote terminal differentiation of precursor cells and cancer cells [38], [39], [40], [41], [42], [43], [44]. We have recently demonstrated that RA signaling plays an important role in regulating myogenic and hepatic progenitor cell differentiation [45], [46]. Nonetheless, our current understanding of the role of RAs in adult stem cells and tissue-specific progenitors is relatively limited. In fact, it remains controversial if RA signaling promotes or inhibits chondrogenic and/or osteogenic differentiation [47], [48], [49], [50], [51], [52], [53], [54].

Here, we investigate the effect of RA signaling on BMP9-induced osteogenic differentiation of MPCs. We find that both 9CRA and ATRA effectively induce early osteogenic marker, such as alkaline phosphatase (ALP), and late osteogenic markers, such as osteopontin (OPN) and osteocalcin (OC). BMP9-induced osteogenic differentiation and mineralization is synergistically enhanced by 9CRA and ATRA *in vitro*. 9CRA and ATRA are shown to induce BMP9 expression and activate BMPR Smad-mediated transcription activity. Using mouse neonatal limb explants, we find that BMP9 and RAs act together to promote the expansion of hypertrophic chondrocyte zone at growth plate. Stem cell implantation studies reveal that co-expression of BMP9 and RXRα or RARα significantly increases trabecular bone and osteoid matrix formation. These results strongly suggest that RA signaling can effectively augment BMP9-induced osteogenic differentiation of MPCs.

## Materials and Methods

### Cell Culture and Chemicals

HEK293 and C3H10T1/2 cells were from ATCC (Manassas, VA). Cell lines were maintained in the conditions as described [24], [26], [28], [29], [30], [31], [55], [56]. All-trans retinoic acid and 9-cis retinoic acid were obtained from BIOMOL (Plymouth, PA). Retinoids were dissolved in DMSO and aliquots were stored in −80°C. DMSO was used as solvent control. For cell culture treated with retinoids, the medium was changed every 3 days. Unless indicated otherwise, all chemicals were purchased from Sigma-Aldrich or Fisher Scientific.

### Recombinant Adenoviruses Expressing RFP, GFP, BMP9, RARα and RXRα

Recombinant adenoviruses were generated using AdEasy technology as described [24], [25], [30], [57], [58]. The coding regions of human BMP9, human RARα and human RXRα were PCR amplified and cloned into an adenoviral shuttle vector and subsequently used to generate recombinant adenoviruses in HEK293 cells. The resulting adenoviruses were designated as AdBMP9, AdR-RARα, and AdR-RXRα. AdBMP9 also expresses GFP, whereas AdR-RARα and AdR-RXRα express RFP as a marker for monitoring infection efficiency. Analogous adenovirus expressing only monomeric RFP (AdRFP) or GFP (AdGFP) were used as controls [27], [28], [29], [30], [31], [57], [58], [59], [60], [61].

### Isolation of Mouse Embryo Fibroblasts (MEFs)

MEFs were isolated from post coitus day 13.5 mice, as previously described [29], [30], [31]. Each embryo was dissected into 10 ml sterile PBS, voided of its internal organs, and sheared through an 18-gauge syringe in the presence of 1 ml 0.25% trypsin and 1 mM EDTA. After 15 min incubation with gentle shaking at 37°C, DMEM with 10% FCS was added to inactivate trypsin. The cells were plated on 100 mm dishes and incubated for 24 hr at 37°C. Adherent cells were used as MEF cells. Aliquots were kept in a liquid nitrogen tank. All MEFs used in this study were less than five passages.

### RNA Isolation and Quantitative Real-Time RT-PCR (qPCR) Analysis

Total RNA was isolated using TRIZOL Reagents (Invitrogen). Total RNA was used to generate cDNA templates by RT reaction with hexamer and Superscript II RT (Invitrogen). The first strand cDNA products were further diluted 5- to 10-fold and used as PCR templates. Quantitative real-time PCR was carried out as described [29], [30], [31], [45], [46], [55], [61], [62]. PCR primers (**Table S1**) were designed by using the Primer3 program to amplify the genes of interest (approximately 150–180 bp). SYBR Green-based qPCR analysis was carried out by using the Opticon DNA Engine (M J Research). The specificity of each qPCR reaction was verified by melting curve analysis and further confirmed by resolving the PCR products on 1.5% agarose gels. Ten-fold serially diluted pUC19 was used as a standard. Triplicate reactions were carried out for each sample. The cycling program was as: 94°C for 2 min for 1 cycle and 30 cycles at 92°C for 20 s, 57°C for 30 s, and 72°C for 20 s, followed by a plate read at 78°C for each cycle. All samples were run in triplicate and normalized by the endogenous expression level of GAPDH.

### Alkaline Phosphatase (ALP) Assay

ALP activity was assessed by a modified Great Escape SEAP Chemiluminescence assay (BD Clontech, Mountain View, CA) and/or histochemical staining assay (using a mixture of 0.1 mg/ml napthol AS-MX phosphate and 0.6 mg/ml Fast Blue BB salt) as described [24], [25], [27], [28], [29], [30], [31], [55], [61]. For the bioluminescence assays, each assay condition was performed in triplicate and the results were repeated in at least three independent experiments. ALP activity was normalized by total cellular protein concentrations among the samples.

### Transfection and Luciferase Reporter Assay

Exponentially growing cells were seeded in 25 cm^2^ cell culture flasks and transfected with 2 µg per flask of BMP receptor Smad-responsive luciferase reporter [63], p12xSBE-Luc or osteocalcin promoter reporter that contains 6 copies of Runx2 responsive elements, p6xOSE-Luc [64], using LipofectAmine (Invitrogen). At 16 hr after transfection, cells were replated to 24-well plates and treated with of 9-cis RA (20 µM), all-trans RA (20 µM) or solvent control. At 48 hr after treatment, cells were lysed and cell lysates were collected for luciferase assays using Promega's Luciferase Assay Kit. Each assay condition was performed in triplicate. The results were repeated in at least three independent experiments. Luciferase activity was normalized by total cellular protein concentrations among the samples. Reporter activity was expressed as mean ± S.D.

### Matrix Mineralization Assay (Alizarin Red S Staining)

C3H10T1/2 cells and MEFs were seeded in 24-well cell culture plates and infected with AdGFP, AdBMP9, and/or RAs. Infected cells were cultured in the presence of ascorbic acid (50 µg/mL) and β-glycerophosphate (10 mM). At 14 days after infection, mineralized matrix nodules were stained for calcium precipitation by means of Alizarin Red S staining, as described previously [24], [25], [27], [28], [29], [30], [31], [55], [61]. Cells were fixed with 0.05% (v/v) glutaraldehyde at room temperature for 10 min. After being washed with distilled water, fixed cells were incubated with 0.4% Alizarin Red S (Sigma-Aldrich) for 5 min, followed by extensive washing with distilled water. The staining of calcium mineral deposits was recorded under bright field microscopy.

### Western Blotting Analysis

Western blotting was performed as previously described [24], [25], [27], [28], [29], [30], [31], [55], [61]. Briefly, cells were collected and lysed in Laemmli buffer. Cleared total cell lysate was denatured by boiling and loaded onto a 4–20% gradient SDS–PAGE. After electrophoretic separation, proteins were transferred to an Immobilon-P membrane. Membrane was blocked with SuperBlock Blocking Buffer, and probed with the primary antibody, anti-osteopontin, anti-osteocalcin, and anti-β-actin (Santa Cruz Biotechnology, Santa Cruz, CA), followed by incubation with a secondary antibody conjugated with horseradish peroxidase. The proteins of interest were detected by using SuperSignal West Pico Chemiluminescent Substrate kit.

### Subcutaneous Progenitor Cell Implantation

The reported work was conducted according to the animal care and use guidelines as stipulated in our protocol No. 71108, which was approved by the Institutional Animal Care and Use Committee of The University of Chicago. The ectopic bone formation by progenitor cell implantation was conducted as described [25], [29], [30], [31], [55]. MEFs were infected with adenoviruses as indicated (MOI = 10). At 16 hr post infection, cells were harvested, and resuspended in PBS for subcutaneous injection (5×10^6^/injection) into the flanks of athymic nude (nu/nu) mice (5 mice per group, 4–6 week old, male, Harlan Sprague Dawley). At 4 wk after implantation, animals were sacrificed, and the implantation sites were retrieved for histologic evaluation, and other stains.

### Fetal Limb Explant Culture

The skinned forelimbs of mouse embryos (E18.5) were dissected under sterile conditions and incubated in DMEM (Invitrogen) containing 0.5% bovine serum albumin (BSA, Sigma), 50 ug/ml ascorbic acid (Sigma), 1 mM β-glycerophosphate and 100 ug/ml penicillin-streptomycin (Mediatech) solution at 37°C in humidified air with 5% CO_2_ for up to 14 days. The limb explants were directly infected by AdBMP9 or AdGFP one day after dissection, followed by 9-cis RA (20 µM), all-trans RA (20 µM), or solvent control treatment. Medium was changed to half volume at day 7. Cultured tissues were observed in different time points under microscope to confirm the survival of tissue cells and the expression of fluorescence markers.

### Histological Evaluation and Trichrome Staining

Retrieved and cultured tissues were fixed in 10% formalin (decalcified with Fisher's Cal-Ex II fixative decalcifier solution if necessary) and embedded in paraffin. Serial sections of the paraffin-embedded samples were deparaffinized, then rehydrated in a graduated fashion, and stained with hematoxylin and eosin (H & E) and Masson's Trichrome [25], [29], [30], [31], [55].

### Statistical Analysis

Microsoft Excel was used to calculate standard deviations (SD) and statistically significant differences between samples using the two-tailed Student's t-test. For all quantitative assays, each assay condition was performed in triplicate and the results were repeated in at least two independent experiments. A *p-value <0.05* was defined as statistically significance.

## Results

### Retinoic acids induce osteogenic differentiation of MPCs

**Figure 1:**
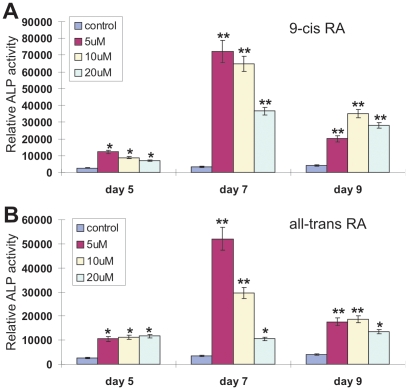
Retinoic acids induce osteogenic differentiation of mesenchymal progenitor cells (MPCs). (**A**) 9-Cis-retinoic acid (9CRA) induces ALP activity in MPCs. Subconfluent C3H10T1/2 cells were treated with varying concentrations of 9CRA or solvent control. ALP activity was measured at the indicated time points. Each assay condition was carried out in triplicate in at least two independent batches of experiments. “*”, *p<0.05*; “**”, *p<0.001* (vs. control groups). (**B**) All-trans-retinoic acid (ATRA) induces ALP activity in MPCs. C3H10T1/2 cells were treated with varying concentrations of ATRA or solvent control. ALP activity was measured at the indicated time points. Each assay condition was carried out in triplicate in at least two independent batches of experiments. “*”, *p<0.01*; “**”, *p<0.001* (vs. control groups).

There have been several conflicting reports about the role of retinoic acids in osteogenic differentiation [47], [48], [49], [50], [51], [52], [53], [54]. Here, we first tested if 9CRA and ATRA can induce osteogenic differentiation in MPCs. By treating the commonly-used MPC line C3H10T1/2 cells with 9CRA (0, 5, 10 and 20 µM), we found that the early osteogenic marker alkaline phosphatase (ALP) activity was significantly induced at as early as at day 5 (**Fig. 1A**). Under the similar conditions, we found that ATRA was able to effectively induce ALP activity (**Fig. 1B**). Interestingly, in either 9CRA or ATRA treated MPCs ALP activity peaked at day 7. However, ALP activity seemingly decreased with increased RA concentrations while the cause of this phenomenon remains to be understood. Similar results were also obtained by using MEFs (data not shown). Collectively, these results demonstrate that 9CRA and ATRA can effectively induce the early osteogenic marker ALP activity in MPCs.

### Retinoic acids and BMP9 act synergistically in inducing ALP activity in MPCs

**Figure 2:**
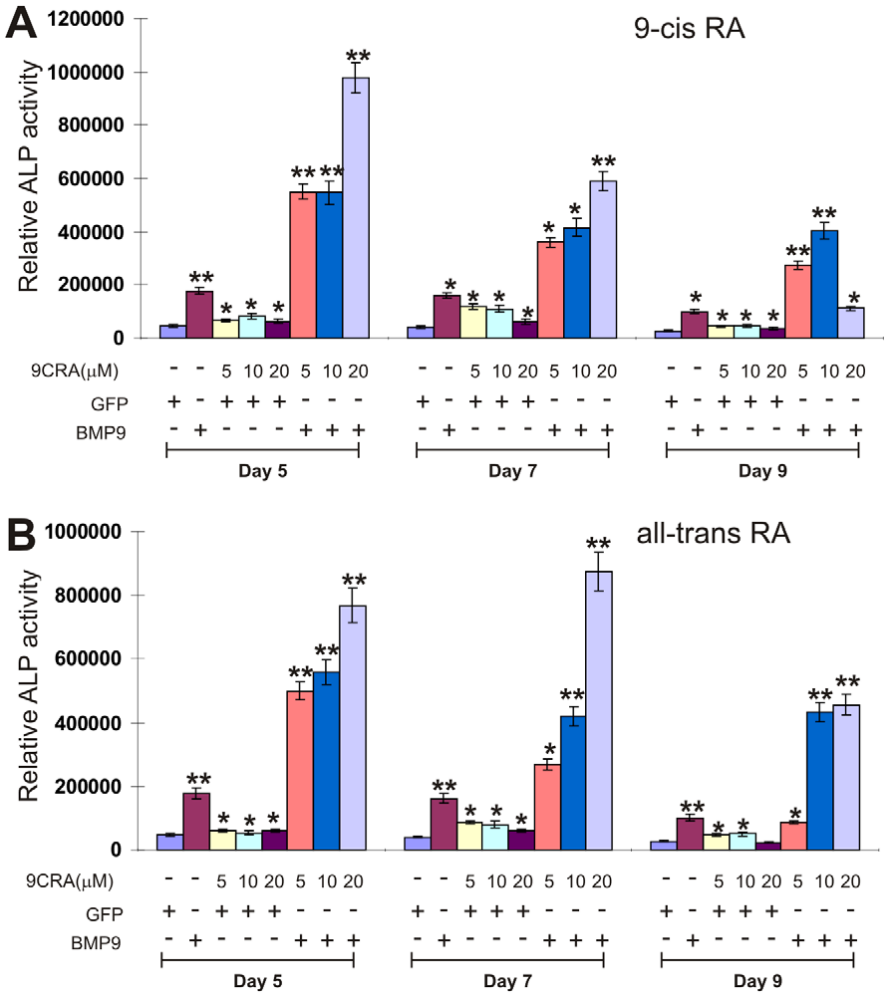
Retinoic acids and BMP9 act synergistically in inducing ALP activity in MPCs. (**A**) 9CRA induces ALP activity in MPCs. Subconfluent MEFs were infected with AdBMP9 (MOI = 5) or AdGFP (MOI = 5), followed by treatment with varying concentrations of 9CRA or solvent control. ALP activity was measured at the indicated time points. Each assay condition was carried out in triplicate in at least two independent batches of experiments. “*”, *p<0.05*; “**”, *p<0.001* (vs. control groups). (**B**) ATRA induces ALP activity in MPCs. MEFs were infected with AdBMP9 (MOI = 5) or AdGFP (MOI = 5), followed by treatment with varying concentrations of ATRA or solvent control. ALP activity was measured at the indicated time points. Each assay condition was carried out in triplicate in at least two independent batches of experiments. “*”, *p<0.05*; “**”, *p<0.001* (vs. control groups).

We next tested if retinoic acids exert any effect on BMP9-induced osteogenic differentiation. We have demonstrated that BMP9 is one of the most potent osteogenic BMPs [5], [19], [24], [25], [27], [28], [29], [30], [31], [55], [61], [65], [66]. When the AdBMP9 or AdGFP-infected MEFs were treated with different concentrations of 9CRA (0, 5, 10 and 20 µM) for 5, 7 and 9 days, we found that 9CRA was able to significantly promote BMP9-induced ALP activity mostly in a dose-dependent manner (**Fig. 2A**). Consistent with the synergistic effect between BMP9 and 9CRA, the ALP activity peaked on day 5, as opposed to day 7 by 9CRA alone (**Fig. 2A** and **Fig. 1A**). Likewise, we also found that ATRA was able to significantly enhance BMP9-induced ALP activity in a dose-dependent manner (**Fig. 2B**). In fact, a combination of BMP9 transduction and RA treatment results in up to 4-fold increase in ALP activity in MEFs. Similar results were also obtained by using C3H10T1/2 cells (data not shown). These results strongly suggest that retinoic acids and BMP9 act synergistically in promoting osteogenic differentiation in MPCs.

### Retinoids potentiate BMP9-induced late osteogenic markers and matrix mineralization in MPCs

**Figure 3:**
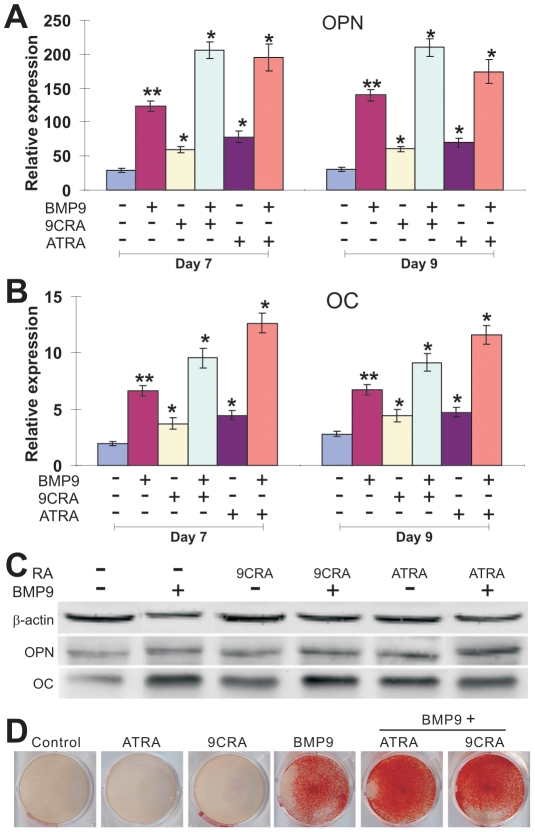
Retinoids potentiate BMP9-induced late osteogenic markers and matrix mineralization in MPCs. (**A**) qPCR analysis of retinoic acids and BMP9 induced osteopontin (OPN) expression. Subconfluent MEFs were infected with AdBMP9 or AdGFP (i.e., -BMP9 groups, MOI = 5), and then treated with 9CRA (20 µM), ATRA (20 µM), or solvent control. At day 7 and day 9, the cells were collected for total RNA isolation. RNA was subjected to RT-PCR transcription, which was used as templates for qPCR analysis using primers specific for mouse OPN. Each assay condition was carried out in triplicate. All samples were normalized using endogenous levels of GAPDH. “*”, *p<0.05*; “**”, *p<0.01* (vs. control groups). (**B**) qPCR analysis of retinoic acids and BMP9 induced osteocalcin (OC) expression. Samples prepared in (A) were used for qPCR analysis using primers specific for mouse OC. Each assay condition was carried out in triplicate. “*”, *p<0.05*; “**”, *p<0.01* (vs. control groups). (**C**) Western blotting analysis of retinoic acids and BMP9 induced OPN and OC expression. MEFs were infected with AdBMP9 or AdGFP (i.e., -BMP9 groups, MOI = 5), and then treated with 9CRA (20 µM), ATRA (20 µM), or solvent control. At day 7, cells were lysed and subjected to Western blotting analysis using anti-OPN or anti-OC antibody (Santa Cruz Biotechnology). Anti-β actin antibody was used to demonstrate equal loading of all samples. (**D**) Retinoic acids and BMP9 induce matrix mineralization. MEFs were infected with AdBMP9 or AdGFP (i.e., -BMP9 groups, MOI = 5), and then treated with 9CRA (20 µM), ATRA (20 µM), or solvent control. At day 14, cells were subjected to Alizarin Red S staining. Experiments were carried out in duplicate and representative results are shown.

We sought to determine if retinoic acids have any effect on BMP9-induced expression of the well-established late osteogenic markers, such as osteopontin (OPN) and osteocalcin (OC) [5], [19], [24], [25], [27], [28], [29], [30], [31], [55], [61], [66]. We infected MEFs with AdBMP9 or AdGFP and then treated with 9CRA (20 µM), ATRA (20 µM), or solvent control for 7 or 9 days, and isolated the total RNA from the cells for quantitative real-time PCR (qPCR) analysis. We found that at both time points a combinations of BMP9/9CRA or BMP9/ATRA treatment resulted in a significant increase in OPN expression (*p<0.05*) (**Fig. 3A**). Likewise, we found that BMP9/9CRA or BMP9/ATRA treatment led to a significant increase in OC expression (*p<0.05*) (**Fig. 3B**). Furthermore, we conducted Western blotting experiments and confirmed that retinoic acids were able to enhance BMP9-induced OPN and OC expression at protein level (**Fig. 3C**). Lastly, we examined the ability of retinoic acids to potentiate BMP9-induced matrix mineralization. As shown in **Fig. 3D**, 9CRA and ATRA were able to augment BMP9-induced mineralization as judged by Alizarin Red S staining. Taken together, the above results strongly suggest that retinoic acids may effectively enhance both early and late stages of BMP9-induced osteogenic differentiation of MPCs.

### Retinoids induce BMP9 expression and activate BMPR-Smad pathway

**Figure 4:**
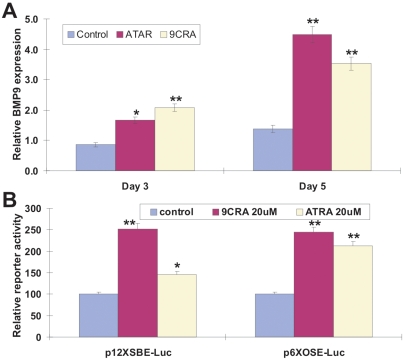
Retinoids induce BMP9 expression and activate BMP-Smad pathway. (**A**) Retinods induce BMP9 expression in MPCs. Subconfluent MEFs were treated with 9CRA (20 µM), ATRA (20 µM), or solvent control. At day 3 and day 5, the cells were collected for total RNA isolation. RNA was subjected to RT-PCR transcription, which was used as templates for qPCR analysis using primers specific for mouse BMP9. Each assay condition was carried out in triplicate. All samples were normalized using endogenous levels of GAPDH. “*”, *p<0.05*; “**”, *p<0.01* (vs. control groups). (**B**) Retinods activate BMPR Smad reporter and Runx2 reporter activity in MPCs. MEFs were transfected with BMPR Smad reporter, p12xSBE-Luc or OC promoter containing Runx2-responsive element reporter, p6xOSE-Luc. The transfected cells were replated at 16h after transfection, followed by a treatment with 9CRA (20 µM), ATRA (20 µM), or solvent control. 48 h after treatment, the cells were lyzed for luciferase activity assay using Promega's Luciferase Assay kit. Each assay condition was carried out in triplicate. Luciferase activity was normalized by total cellular protein concentrations among the samples. “*”, *p<0.05*; “**”, *p<0.001* (vs. control groups).

We next sought to explore the possible mechanism behind the synergy between retinoic acids and BMP9 in osteogenic differentiation. It has been reported that retinoids may regulate BMP2 and/or BMP4 expression [67], [68], [69], [70], [71]. As BMP9 is one of the least studied BMPs until recently [5], [19], [24], [25], [27], [28], [29], [30], [31], [55], [66], the regulation of BMP9 expression is not well understood. Here, we treated MEFs with 9CRA (20 µM), ATRA (20 µM), or solvent control for 3 and 5 days. Using qPCR analysis, we found that retinoic acids were able to significantly up-regulate BMP9 expression (*p<0.05*) (**Fig. 4A**).

We further examined the effect of retinoic acids on Smad signaling and Runx2-related gene regulation. Using the BMPR Smad reporter p12xSBE-Luc [63], we found that both 9CRA and ATRA were able to enhance the BMPR Smad reporter activity by approximately 150% and 50%, respectively (*p<0.05*) (**Fig. 4B**, left panel). Likewise, 9CRA and ATRA were able to increase the osteocalcin promoter reporter activity, which contains Runx2-responsive elements and reflects Runx2-regulated osteocalcin expression [64], by approximately 150% and 100%, respectively (*p<0.05*) (**Fig. 4B**, right panel). Collectively, these results suggest that retinoic acids may exert synergistic effect at least in part through up-regulation of BMP9 expression and activation of BMPR-Smad signaling axis.

### Retinoids and BMP9 promote the expansion of hypertrophic chondrocyte zone in fetal limb culture

**Figure 5:**
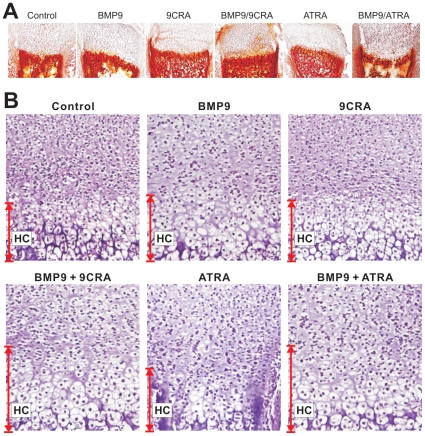
Retinoids and BMP9 promote the expansion of hypertrophic chondrocyte zone in organ culture. (**A**) Harvest, transduction and labeling of mouse E18.5 forelimbs (n = 5 each group). E18.5 forelimbs were dissected, and the skin was removed with the soft tissues attached. Recombinant adenovirus (5×10∧10 pfu in 1 ml medium) expressing AdGFP or AdBMP9 added to culture medium, with or without 9CRA (20 µM) or ATRA (20 µM). After two weeks, the cultured limbs were harvested, embedded, and subjected to Alizarin Red S stating. Representative low magnification images are shown. (**B**) Histologic evaluation. The above samples were subjected to sectioning and H & E staining, and recorded under bright field with 400× magnification. The approximate lengths of hypertrophic zones were indicated. Representative images are shown. HC, hypertrophic chondrocyte zone.

To further investigate the synergy between retinoic acids and BMP9, we conducted tissue explant experiments. Using mouse E18.5 perinatal forelimbs, we found that a combination of BMP9 transduction and retinoic acid treatment led to a significant expansion of the hypertrophic chondrocyte zone of the cultured fetal limbs (**Fig. 5A** and **5B**). It is noteworthy that we did not observe any significant increases in the overall length of the cultured limbs. However, this may be at least in part due to the limited culture duration (e.g., <2 weeks) so that the overall ossification and limb growth were not significantly affected. Nonetheless, these organ culture experiments suggest that BMP9 and 9CRA or ATRA may act synergistically to accelerate chondrocyte hypertrophy and subsequently endochrondral bone formation.

### Overexpression of retinoic acid receptors enhance BMP9-induced ectopic bone formation from MPCs

Lastly, we sought to test the effect of retinoids on BMP9-induced *de novo* ectopic bone formation from MPCs. We have demonstrated that ectopic bone formation by stem cell implantation is a reliable and effective approach [25], [29], [30], [31]. In order to avoid the challenging deliveries of 9CRA and ATRA for *in vivo* studies, we have recently constructed adenoviral vectors expressing RARα and RXRα receptors, and demonstrated that they are constitutively active in a ligand-independent fashion when they are over-expressed [44], [45], [46].

**Figure 6:**
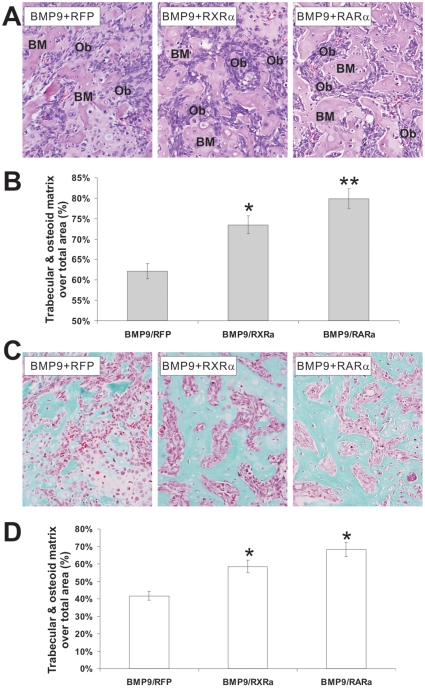
Retinoids enhance BMP9-induced ectopic ossification. (**A**) Histological evaluation of ectopic bone formation. Subconfluent MEFs were co-infected with AdBMP9 and AdRFP, AdR-RXRα, or AdR-RARα adenoviruses (MOI = 10) for 16 h. The infected cells were implanted subcutaneously (5×10^6^/injection) into the flanks of athymic nude (nu/nu) mice (5 mice/group, 4–6 week old, male, Harlan Sprague Dawley). At 4 wk after implantation, animals were sacrificed, and the implantation sites were retrieved, fixed and decalcified. The paraffin-embedded sections were subjected to H & E staining. Representative images are shown. BM, bone matrix (ossified and osteoid); Ob, osteoblast. (**B**) Quantitative analysis of trabecular and osteoid matrix area. The average trabecular bone and osteoid matrix areas were determined. At least 10 samples (with ×100 magnification) from each group were randomly selected and analyzed by using ImageJ software. “*” *p<0.05*, “**” *p<0.001*. (**C**) Masson's Trichrome staining of ectopic bone masses. Tissue sections prepared in were subjected to Masson's Trichrome staining. Representative images are shown. Magnification, ×400. (**D**) Quantitative analysis of % trabecular/osteoid area over total area was done by using ImageJ. At least 10 samples (with ×100 magnification) from each group were randomly selected and analyzed. “*” *p<0.01*.

We co-infected MEFs with AdBMP9 and AdR-RARα, AdRXRα, or AdRFP control and implanted the cells subcutaneously in the flanks of athymic nude mice for four weeks. Although we did not observe any significant differences in the overall sizes of bony masses among the groups, histologic analysis of the retrieved bone masses indicated that the trabecular bone and osteoid matrix areas increased significantly in BMP9/RXRα and BMP9/RARα-transduced groups (*p<0.05*) (**Figs. 6A** and **6B**). It is noteworthy that AdRFP alone, AdRFP/AdR-RXRα and AdRFP/AdR-RARα groups did not form any discernable bone masses. Using Masson's Trichrome staining, we further analyzed the difference in trabecular bone and osteoid areas among the groups, and found that BMP9/RXRα and BMP9/RARα-transduced groups exhibited a significant increase in trabecular bone and osteoid areas (*p<0.01*) (**Figs. 6C** and **6D**). These *in vivo* results strongly suggest that retinoid signaling may synergize with BMP9 activity in promoting osteogenic differentiation of MPCs.

## Discussion

We have demonstrated that BMP9 is one of the most potent osteogenic BMPs [19], [24], [25], [26], [27], [28], [29], [30], [31]. Yet BMP9 remains as one of the least studied BMPs. BMP9 (also known as growth differentiation factor 2, or GDF-2) was first identified in developing mouse liver [72], and its possible roles include inducing and maintaining the cholinergic phenotype of embryonic basal forebrain cholinergic neurons [73], inhibiting hepatic glucose production and inducing the expression of key enzymes of lipid metabolism [74], and stimulating murine hepcidin 1 expression [75]. Although the functional role of BMP9 in skeletal system remains to be fully understood, its potent osteogenic activity suggests that it may be used as an efficacious bone regeneration agent. It is conceivable that other signaling molecules may act synergistically to enhance BMP9-induced bone formation.

In this report we investigate the effect of RA signaling on BMP9-induced osteogenic differentiation of MPCs. We find that both 9CRA and ATRA effectively induce early osteogenic marker and late osteogenic markers. BMP9-induced osteogenic differentiation and mineralization is synergistically enhanced by 9CRA and ATRA *in vitro*. 9CRA and ATRA are shown to induce BMP9 expression and activate BMPR Smad-mediated transcription activity. Using mouse perinatal limb explants, we find that BMP9 and RAs act together to promote the expansion of hypertrophic chondrocyte zone at growth plate. Stem cell implantation studies reveal that co-expression of BMP9 and RXRα or RARα significantly increases trabecular bone and osteoid matrix formation. These results strongly suggest that RA signaling can effectively augment BMP9-induced osteogenic differentiation of MPCs.

Retinoids play an important role in embryonic development and function maintenance of vital organs in adult [32], [33]. Retinoic acid is formed solely from retinaldehyde (Rald), which is derived from vitamin A. The metabolism of vitamin A and the diverse effects of its metabolites are tightly controlled by distinct retinoid-generating enzymes, retinoid-binding proteins and retinoid-activated nuclear receptors [32], [76]. RA regulates differentiation and metabolism by serving as a ligand for two families of nuclear receptors RARs that bind the abundant form of RA known as ATRA, and the RXRs that bind an isomer known as 9CRA [34], [35], normally undetectable except when vitamin A is present in excess [36]. RXR forms heterodimers with RAR and several other nuclear receptors when bound to DNA, suggesting that RXR may function as a scaffold protein to facilitate DNA binding for several types of nuclear receptors [34], [35]. In vivo studies have demonstrated that ligand binding to just the RAR portion of RAR/RXR heterodimers is sufficient and necessary to rescue a lethal defect in RA synthesis, whereas ligand binding to RXR does not rescue the defect and is unnecessary [36]. RA-induced transcriptional activity is tightly regulated by nuclear co-repressors (NCORs) and nuclear receptor co-activators (NCOAs) [77]. Genetic manipulations in animals have revealed that RA signaling is important for the development of the forebrain and the segmented hindbrain, and for the elongation of the body axis [32], [33], [35]. RA signaling has also been implicated in early heart patterning, forelimb induction, pancreas induction, lung induction, eye formation, and some aspects of genitourinary tract development [32], [33], [78]. However, our current understanding of the role of RA in adult stem cells, and tissue-specific progenitors is relatively limited.

While conflicting results have been reported about RA's role in BMP-induced osteogenic differentiation, it is well established that RA plays an important role in differentiation and bone metabolism. Rogers et al showed that BMP2 and BMP4 were involved in the retinoic acid-induced differentiation of embryonal carcinoma cells [70]. Targeted disruption of RARα and RARγ resulted in receptor-specific alterations in retinoic acid-mediated differentiation and retinoic acid metabolism [67]. BMP2 and RA signaling may cooperate to stimulate cell proliferation, repress adipogenesis, and promote osteoblast differentiation of preadipocytes [53]. Retinoic acid stimulated chondrocyte differentiation and enhanced BMP2 effects through induction of Smad1 and Smad5 [69]. Cowan et al demonstrated that BMP2 and RA accelerate *in vivo* bone formation, osteoclast recruitment, and bone turnover [51]. Using mouse adipose-derived adult stromal cells, Wan et al demonstrated that osteogenic differentiation requires retinoic acid and BMP receptor type IB signaling [48]. Interestingly, Hoffman et al reported that BMP4 action in skeletogenesis involved attenuation of retinoid signaling [49]. Wang et al recently showed that ATRA inhibited osteogenic differentiation of rat bone marrow stromal cells [47]. Currently, no satisfactory explanations about the conflicting observations can be offered, although it is conceivable that the synergistic action of RA and BMP may depend on different stages of osteoblastic differentiation of MPCs. Nonetheless, we have demonstrated that RA can promote BMP9-induced osteogenic differentiation and bone formation.

It has well established that RA signaling plays an important role in chondrogenesis. Cash et al reported that overexpression of RARα in transgenic animals interfered with chondrogenesis and leads to appendicular skeletal defects [79]. Further analysis of these animals showed that expression of the transgene in chondroprogenitors maintained a prechondrogenic phenotype and prevented chondroblast differentiation [54]. However, RA has been shown to stimulate chondrocyte differentiation and enhances BMP2 effects [69]. Interestingly, both RA and BMP2 induced expression of the chondrocyte maturational marker colX in chondrocyte cultures. Though the RA effect was small, it synergistically enhanced the effect of BMP2 on colX and ALP activity [69]. BMP2 did not enhance the effects of RA on an RA-responsive reporter, but RA enhanced basal activity and synergistically enhanced BMP2 stimulation of the BMP-responsive type X collagen reporter [69]. Drissi et al showed that Runx2 stimulation by RA is potentiated by BMP2 signaling through interaction with Smad1 on the collagen X promoter in chondrocytes [52]. Consistent with the above observation, it has been recently reported that retinoids directly activate the collagen X promoter in prehypertrophic chondrocytes through a distal retinoic acid response element [50]. In our studies, we have found that BMP9 and RA act together to promote the expansion of hypertrophic chondrocyte zone in perinatal limb culture.

Mechanistically, it remains to be thoroughly investigated how RA signaling cooperates with BMP9 pathway in osteogenesis. We have shown that 9CRA and ATRA can induce BMP9 expression and activate BMPR Smad-mediated transcription activity. Li et al showed that RA did not increase the expression of the type IA BMP receptor but did markedly up-regulate the expression of Smad1 and Smad5 proteins and that inhibition of RA signaling, with the selective inhibitor AGN 193109, blocked RA-mediated induction of the Smad proteins and chondrocyte differentiation [69]. Hatakeyama et al reported that expression of BMP2 mRNA was stimulated by retinoic acid in human adenocarcinoma cell line HSG-S8 [68]. The absence of RARγ was associated with a loss of the RA-inducible expression of the collagen IV (alpha 1) and BMP2 genes [67]. Nonetheless, the molecular mechanism behind the cross-talk of RA signaling and BMP9 pathway requires further investigation.

In summary, we find that retinoids effectively induce early osteogenic marker and late osteogenic markers. BMP9-induced osteogenic differentiation and mineralization is synergistically enhanced by RA in vitro. Retinoinds are shown to induce BMP9 expression and to activate BMPR Smad-mediated transcription activity. We find that BMP9 and RAs act together to promote the expansion of hypertrophic chondrocyte zone at the growth plate of mouse perinatal limb explants. Stem cell implantation studies reveal that co-expression of BMP9 and RXRα or RARα significantly increases trabecular bone and osteoid matrix formation. These results strongly suggest that RA signaling can effectively augment BMP9-induced osteogenic differentiation of MPCs. This knowledge should expand our understanding about how BMP9 cross-talks with other signaling pathways. Furthermore, a combination of BMP9 and retinoic acid (or its agonists) may be explored as effective bone regeneration therapeutics to treat large segmental bony defects, non-union fracture, and/or osteoporotic fracture.

## Supporting Information

Table S1 (0.02 MB XLS)
